# The hand of evolution: conservation and divergence of *hand* paralogs in cyclostomes and gnathostomes

**DOI:** 10.3389/fcell.2026.1944613

**Published:** 2026-10-29

**Authors:** Galina V. Ermakova, Irina V. Meyntser, Alexandr V. Kucheryavyy, Andrey G. Zaraisky, Andrey V. Bayramov

**Affiliations:** 1 Shemyakin-Ovchinnikov Institute of Bioorganic Chemistry, Russian Academy of Sciences, Moscow, Russia; 2 Koltzov Institute of Developmental Biology, Russian Academy of Sciences, Moscow, Russia; 3 Mosqvarium Center for Oceanography and Marine Biology, Moscow, Russia; 4 Severtsov Institute of Ecology and Evolution, Russian Academy of Sciences, Moscow, Russia

**Keywords:** branchial arches, cyclostomes, fins, gnathostomes, hand, lamprey, paired appendages, shark

## Abstract

The Hand family comprises basic helix-loop-helix transcription factors with key roles in the development of the lateral mesoderm, heart, branchial arches and paired appendages in vertebrates. Although Hand1 and Hand2 have been extensively studied in model vertebrates, the evolutionary history and diversification of *Hand* paralogs in early-diverging vertebrate lineages remain poorly understood. Here, we combined phylogenetic reconstruction, local genomic synteny analysis, and spatiotemporal expression profiling to investigate *Hand* paralogs in the lamprey *Lampetra fluviatilis* and the cartilaginous fish *Chiloscyllium griseum*. Phylogenetic analyses resolve the correspondence of 3 *L. fluviatilis* paralogs - *handα*, *handβ*, and *handγ* - with their *Petromyzon marinus* counterparts, whereas deeper relationships between cyclostome *Hand* paralogs and gnathostome *hand1*/*hand2* remain unresolved; local genomic synteny provides complementary but insufficient evidence for one-to-one orthology across the cyclostome–gnathostome split. During lamprey embryogenesis, *handα* and *handβ* show partially overlapping but distinct spatiotemporal expression patterns, including a previously unrecognized *handβ* domain in the caudal fin, whereas no specific *handγ* signal was detected under the conditions examined. In *C. griseum*, *hand1* and *hand2* show distinct expression patterns in the branchial apparatus and developing fins: *hand2* is expressed in posterior domains of paired and dorsal fins, whereas *hand1* is detected predominantly in paired fins by whole-mount in situ hybridization, with substantially lower transcript abundance in dorsal fins supported by targeted qRT-PCR. Together, these findings resolve distinct cyclostome *Hand* paralogs and reveal lineage- and paralog-specific diversification of *Hand* expression while supporting an evolutionarily ancient deployment of *Hand* genes in the vertebrate branchial region and fin-associated developmental programs.

## Introduction

Compared with their invertebrate chordate relatives, cephalochordates and tunicates, vertebrates possess several evolutionary innovations, including neural crest cells, cranial placodes, a complex cranium, and paired appendages, that contributed to the morphological complexity of the vertebrate body plan (Northcutt and Gans, 1983; Shimeld and Holland, 2000; Holland and Holland, 2001; Holland et al., 2008; Bronner and LeDouarin, 2012; Martik and Bronner, 2021). The diversification of vertebrate morphology was likely facilitated by whole-genome duplications (WGDs) early in vertebrate evolution, which expanded developmental gene repertoires and contributed to increasingly complex gene regulatory networks (Dehal and Boore, 2005; Marlétaz et al., 2024; Yu et al., 2024). Current evidence indicates that an ancestral vertebrate WGD was followed by independent polyploidization events in the cyclostome and gnathostome lineages (Marlétaz et al., 2024; Yu et al., 2024). These duplications generated multiple paralogs of developmental regulators, including members of the *Hox*, *Shh*, *Fgf*, and *Hand* families (Holland and Ocampo Daza, 2018; Sacerdot et al., 2018; Reynolds et al., 2021). Comparative analyses of early-diverging vertebrates, such as lampreys (Cyclostomata) and cartilaginous fishes (Chondrichthyes), are therefore essential for reconstructing the evolutionary divergence of duplicated genes (Smith et al., 2018; Venkatesh et al., 2014).

Paired appendages are a major morphological innovation of gnathostomes (Cass et al., 2021; Bayramov et al., 2024). Among their key developmental regulators are the *Hand* (heart and neural crest derivatives expressed) genes (Firulli, 2003; Reynolds et al., 2021). These genes encode basic helix–loop–helix (bHLH) transcription factors involved in the development of the lateral mesoderm, heart, and neural crest-derived structures (Firulli, 2003; Barnes et al., 2010). Most gnathostomes possess two paralogs, *hand1* and *hand2*, derived from genome duplication events (Firulli, 2003; Reynolds et al., 2021). However, the number and genomic organization of *Hand* genes vary among vertebrate lineages, making this family a useful model for examining the evolutionary consequences of gene duplication (Angelo et al., 2000; Ochi et al., 2017; Reynolds et al., 2021).

In amphioxus, a cephalochordate that retains many ancestral chordate features, a single *AmphiHand* gene has been identified (Putnam et al., 2008; Marlétaz et al., 2018). *AmphiHand* is expressed throughout the ventral mesoderm from the late neurula stage and overlaps with *AmphiNk2-tin*, an ortholog of *Nkx2.5*, suggesting that the cardiac mesoderm (CM) and post-cardiac lateral plate mesoderm (PLPM) are not clearly segregated in this lineage (Onimaru et al., 2011). These observations suggest that the ancestral function of *Hand* genes involved broad patterning of the ventral mesoderm rather than the development of specialized derivatives.

Lamprey genomes underwent lineage-specific polyploidization after the divergence of cyclostomes and gnathostomes (Marlétaz et al., 2024). Earlier studies described a single lamprey *hand* gene that occupied a basal position relative to gnathostome *hand1* and *hand2* (Häming et al., 2011). Unlike amphioxus, lampreys show compartmentalization of the lateral mesoderm into cardiac mesoderm (CM) and posterior lateral plate mesoderm (PLPM) domains. *Hand* expression has been detected in the cardiac region, ventral branchial mesenchyme, posterior mesoderm, lower-lip mesenchyme, and ventral ectomesenchyme of the branchial arches (Onimaru et al., 2011; Cerny et al., 2010; Kuraku et al., 2010). Thus, *Hand* genes appear to have acquired roles in multiple developmental processes before the emergence of gnathostomes.

Cartilaginous fishes, including chimaeras, sharks, and rays, represent the earliest-diverging extant gnathostome lineage and provide important insights into the evolution of developmental regulatory networks in jawed vertebrates (Gillis and Shubin, 2009; Venkatesh et al., 2014; Renz et al., 2013). In the shark *Scyliorhinus canicula*, *hand2* is expressed in the posterior mesenchyme of the pectoral fins and overlaps with components of the Shh-dependent zone of polarizing activity (ZPA) network, consistent with deep conservation of mechanisms controlling anteroposterior patterning of paired appendages (Tanaka et al., 2002; Onimaru et al., 2015). *Hand1* has also been reported in the pectoral fin, with a distribution distinct from *hand2* (Onimaru et al., 2015). However, available data on *Hand* expression in cartilaginous fishes remain largely restricted to pectoral fins, precluding a broader assessment of paralog-specific expression across appendage types.

In gnathostomes, both *Hand* paralogs contribute to craniofacial development, although their functions overlap only partially. *Hand2* is a conserved regulator of ventral identity in the branchial arches of teleosts, birds, and mammals (Thomas et al., 1998; Charité et al., 2000; Yelon et al., 2000; Miller et al., 2003), whereas *Hand1* has more restricted roles in mandibular morphogenesis (Barbosa et al., 2007). Experimental studies have further shown that *Hand2* controls lower-jaw specification by regulating multiple downstream transcription factors and suppressing premature osteogenesis (Funato et al., 2009; 2016). Expression analyses in the cichlid *Copadichromis azureus* likewise revealed distinct spatial domains of *hand1* and *hand2* within the visceral arches, illustrating paralog-specific deployment during craniofacial development (Reynolds et al., 2021).

Hand2 is also a conserved regulator of appendage development across gnathostomes and acts upstream of the Shh-dependent zone of polarizing activity (ZPA) signaling network during anteroposterior patterning (Charité et al., 2000; Yelon et al., 2000; te Welscher et al., 2002; Galli et al., 2010; Osterwalder et al., 2014). In teleosts, *hand2* is expressed in the posterior mesenchyme of paired and median fins and is required for normal pectoral-fin development (Yelon et al., 2000; Reynolds et al., 2021). A related role was recently reported during axolotl limb regeneration, in which *hand2* regulates positional memory and Shh activation in the blastema (Otsuki et al., 2025). By contrast, *hand1* expression is more variable among gnathostomes, and the gene appears to have a comparatively minor or partially redundant role in appendage development, although altered Hand1 phosphorylation disrupts limb morphogenesis in mice (Fernandez-Teran et al., 2003; Howe et al., 2013; Firulli et al., 2017; Reynolds et al., 2021).

Despite extensive studies of *Hand* genes in model organisms, their evolutionary history remains poorly resolved in the two earliest-diverging major vertebrate lineages: cyclostomes and cartilaginous fishes. Lampreys have traditionally been considered to possess a single *hand* gene, whereas *hand* expression data in cartilaginous fishes have focused mainly on pectoral fins. Here, we identify and characterize *hand* paralogs in the river lamprey *Lampetra fluviatilis* and the grey bamboo shark *Chiloscyllium griseum*. By combining phylogenetic reconstruction, local genomic synteny analysis, and expression profiling, we examine the diversification of the *Hand* gene family and the specialization of its paralogs during early vertebrate evolution.

## Results

### Phylogenetic analysis and local genomic synteny of hands in vertebrates

Comparative sequence analysis identified multiple *Hand* paralogs in cyclostomes. Three *Hand* genes, designated *handα*, *handβ* and *handγ*, were identified in the sea lamprey *Petromyzon marinus*, whereas two *Hand* genes corresponding to the *α* and *β* lineages were identified in the hagfish *Myxine glutinosa*. Three corresponding Hand sequences from European river lamprey *L. fluviatilis* were included directly in the phylogenetic analysis. Most examined gnathostome lineages contained representatives of the Hand1 and Hand2 groups, with lineage-specific gene losses and additional duplicated copies in some taxa.

Maximum-likelihood analyses recovered the major gnathostome Hand1 and Hand2 groups (Figure 1). The *L. fluviatilis* Hand sequences examined in this study clustered with the corresponding *P. marinus* Handα, Handβ and Handγ sequences, whereas the *C. griseum* Hand1 and Hand2 sequences were recovered within the gnathostome Hand1 and Hand2 groups, respectively.

**FIGURE 1:**
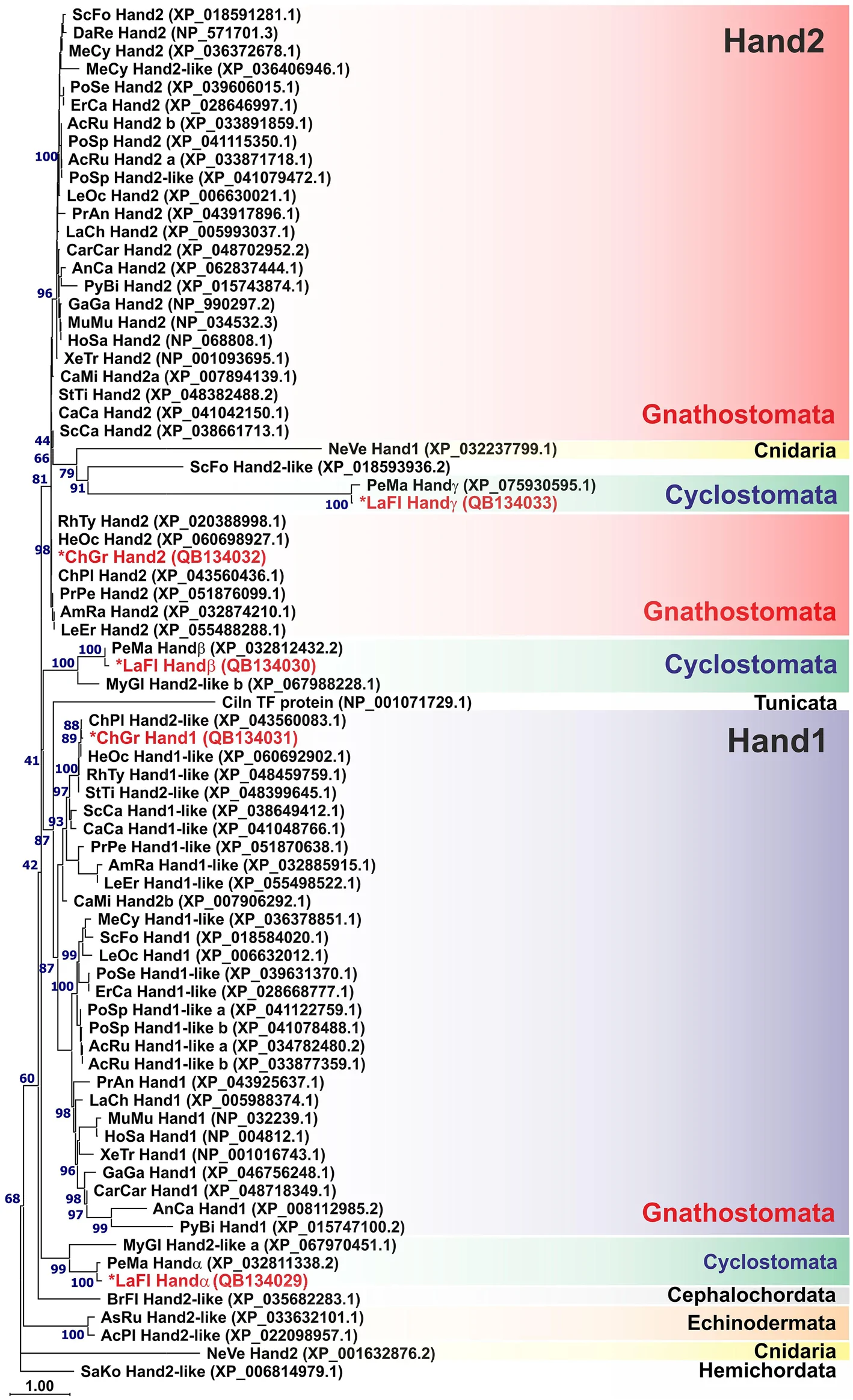
Maximum-likelihood phylogenetic analysis of Hand/Hand-like proteins. The tree was inferred from a 204-amino-acid trimmed alignment using the site-heterogeneous LG + C60 + F + G4/PMSF model in IQ-TREE. The experimentally characterized *L. fluviatilis* Handα, Handβ and Handγ and *C. griseum* Hand1 and Hand2 sequences are shown in red and marked with asterisks. Selected ultrafast bootstrap (UFBoot) support values based on 1,000 replicates are shown at nodes relevant to the placement of the experimentally characterized sequences and representative deeper relationships. Background shading indicates the taxonomic affiliation of the sequences and is used for visualization only; it does not denote phylogenetically supported clades. The Hand-family tree is presented as an unrooted topology because inclusion of a divergent *B*. *floridae* Twist-like bHLH sequence did not provide a reliable biological root in the outgroup-sensitivity analysis (Supplementary Figure S1). Branch lengths represent substitutions per site. Accession numbers are indicated after the sequence names. The complete sequence inventory used for the phylogenetic analyses is provided in Supplementary Table S3. AcPl - *Acanthaster planci*; AcRu - *Acipenser ruthenus*; AmRa - *Ambystoma radiata*; AnCa - *Anolis carolinensis*; AsRu - *Asterias rubens*; BrFl - *Branchiostoma floridae*; CaCa - *Carcharodon carcharias*; CaMi - *Callorhinchus milii*; CarCar - *Caretta*; *ChGr - Chiloscyllium griseum;* ChPl - *Chiloscyllium plagiosum*; DaRe - *Danio rerio*; ErCa - *Erpetoichthys calabaricus*; GaGa - *Gallus gallus*; HeOc - *Hemiscyllium ocellatum*; HoSa - *Homo sapiens*; LaCh - *Latimeria chalumnae*; LaFl - *Lampetra fluviatilis*; LeEr - *Leucoraja erinaceus*; LeOc - *Lepisosteus oculatus*; MeCy - *Megalops cyprinoides*; MuMu - *Mus musculus*; MyGl - *Myxine glutinosa*; NeVe - *Nematostella vectensis*; PeMa - *Petromyzon marinus*; PoSe - *Polypterus senegalus*; PoSp - *Polyodon spathula*; PrAn - *Protopterus annectens*; PrPe - *Pristis pectinata*; PyBi - *Python bivittatus*; RhTy - *Rhincodon typus*; SaKo - *Saccoglossus kowalevskii*; ScCa - *Scyliorhinus canicula*; ScFo - *Scleropages formosus*; StTi - *Stegostoma tigrinum*; XeTr - *Xenopus tropicalis*.

Deeper relationships between cyclostome Hand paralogs and gnathostome Hand1/Hand2 remained weakly resolved and were sensitive to model and outgroup treatment. Inclusion of a divergent *Branchiostoma floridae* Twist-like bHLH sequence as the outgroup did not alter the placement of the *L. fluviatilis* and *C. griseum* sequences examined in this study but did not provide a stable root for the Hand-family tree (Supplementary Figure S1). We therefore treat the principal Hand phylogeny as unrooted.

To test alternative one-to-one orthology scenarios quantitatively, constrained topologies were compared using the approximately unbiased (AU) test. None of the tested placements of cyclostome Handα or Handβ with either gnathostome Hand1 or Hand2 was rejected (AU *P* = 0.121–0.629; Supplementary Table S1). Thus, the sequence data robustly resolve the correspondence of the lamprey α, β and γ copies between *L. fluviatilis* and *P. marinus*, but do not provide sufficient phylogenetic resolution to establish one-to-one orthology between cyclostome and gnathostome *Hand* paralogs.

Local genomic synteny analysis revealed distinct and partially conserved contexts associated with gnathostome *hand1* and *hand2* loci (Supplementary Figure S2). The *hand1* neighborhood frequently included *cnot8*, *faxdc2* and *larp1* together with *sap30L* and *galnt10*, whereas *hand2* loci retained a different set of neighboring genes, including *hpgd*, *cep44*, *fbxo8*, *scrg1*, *sap30* and *galnt7*. The paralogous combinations *hand1–sap30L–galnt10* and *hand2–sap30–galnt7* correspond to duplicated local genomic regions previously analysed in human, mouse and chicken (Viiri et al., 2009).

Cyclostome *P. marinus Hand* loci retained subsets of genes found in gnathostome *Hand* neighborhoods, but their local genomic contexts did not provide unambiguous evidence for one-to-one orthology between cyclostome *handα/β/γ* and gnathostome *hand1/hand2*. Accordingly, local genomic context was treated as complementary evidence to the phylogenetic analysis rather than as an independent reconstruction of *Hand* duplication history.

The recent chromosome-level *L. fluviatilis* assembly kcLamFluv1.1 (GCF_964198595.1; Tørresen et al., 2026) was also examined. Sequence-based searches recovered the three experimentally relevant *Hand* loci, including *handα* on chromosome 15 (OZ243577.1), the annotated *handβ* locus (LOC144937656; XM_078865043.1), and *handγ* on chromosome 5 (OZ243567.1) (Supplementary Table S2). Because annotation of the immediate neighborhoods surrounding the sequence-defined *handα* and *handγ* loci is incomplete, these loci were not represented as graphical synteny rows; absence of individual neighboring genes from the current annotation was not interpreted as evidence of gene loss.

Thus, several *Hand*-like loci are present in cyclostomes, including *handα* and *handβ* in lamprey and hagfish and an additional *handγ* lineage in lampreys. The phylogeny strongly supports α/β/γ correspondence between *L. fluviatilis* and *P. marinus*, whereas the available local genomic synteny remains insufficient to assign these cyclostome paralogs directly to gnathostome *hand1* or *hand2*.

The *handα* neighborhoods in cyclostomes retain subsets of genes also found in the gnathostome *hand2*-associated region, whereas *handβ* occurs in a different local context. These partial correspondences are informative at the local synteny level but do not independently reconstruct ancestral chromosome-scale linkage relationships.

Together, the phylogenetic and local genomic synteny analyses distinguish stable within-cyclostome paralog correspondences while leaving the deeper cyclostome-to-gnathostome one-to-one relationships unresolved.

A single *hand-like* gene was identified in *B. floridae*. It lies adjacent to an *fbxo8-like* gene, whose ortholog is a component of the gnathostome *hand2* neighborhood, and may therefore retain part of an ancestral *hand*-associated genomic region (Supplementary Figure S2).

### Expression of *hand* paralogs during lamprey *L. fluviatilis* embryogenesis

Three *hand* paralogs, designated *handα*, *handβ*, and *handγ*, were identified in the lamprey genome. Their spatial expression patterns were examined by whole-mount *in situ* hybridization (ISH).

*Handα* expression was first detected in scattered cells at stage 13 (Tahara, 1988; Figure 2A1). At the neurula stage (stage 17), *handα*-expressing cells were observed in the ventral and lateral regions of the embryo (Figure 2A2). By the late neurula stage (stage 19), expression was detected bilaterally alongside the neural tube (Figures 2A3, A4). During head-process formation (stages 22–23), *handα* was expressed in the ventral mesenchyme of both the head and trunk (Figures 2A5–A7).

**FIGURE 2:**
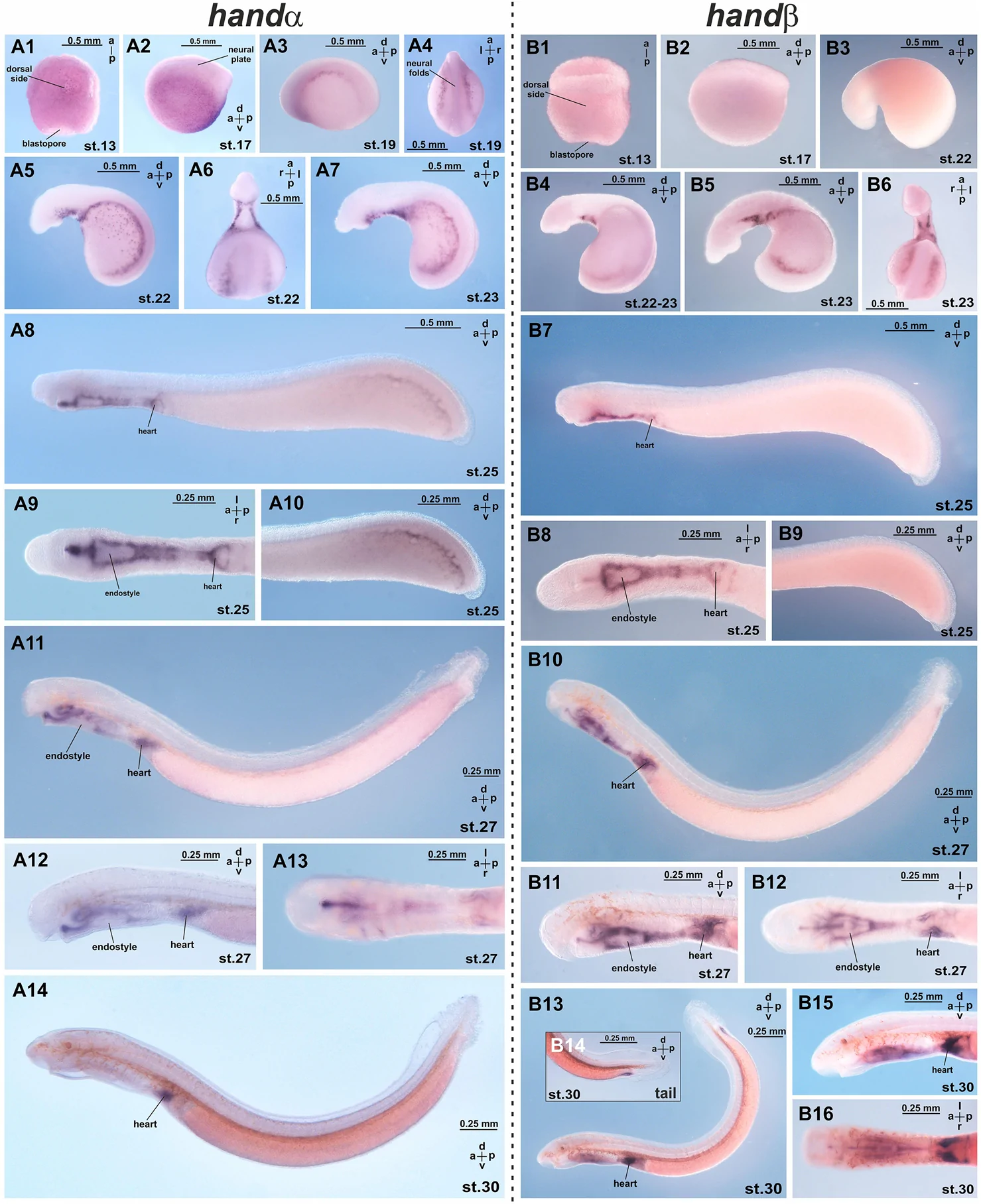
Expression of *handα* and *handβ* in *L*. *fluviatilis* embryos at stages 13–30 (staged according to Tahara, 1988). *Handα* expression was first detected at stage 13 in individual cells **(A1)**. At the neurula stage (stage 17), *handα* expression was observed in cells located in the ventral and lateral regions of the embryo **(A2)**. At the late neurula stage (stage 19), expression was detected in cells located on both sides, lateral to the neural tube **(A3, A4)**. During stages 22–23, *handα* expression was detected in the ventral mesenchyme of both the head and trunk regions **(A5–A7)**. At stage 25, *handα* expression was detected in the ventral mesenchyme surrounding the endostyle in the ventral region of the pharynx, in the medial stripe of the lower lip mesenchyme, in the cardiac region, and in the caudal region **(A8–A10)**. At stage 27, expression was detected in branchial areas and in the heart **(A11–A13)**. At stage 30, handα expression was retained exclusively in the cardiac region **(A14)**.Handβ expression was first detected during stages 22–23 in the ventral mesenchyme of the head outgrowth **(B1–B6)**. At stage 25, handβ expression was detected in the ventral mesenchyme surrounding the endostyle in the ventral region of the pharynx and in the cardiac region **(B7, B8)**. Expression in the medial stripe of the lower lip mesenchyme was also observed, although weaker compared with handα, whereas expression in the caudal region was not detected **(B9)**. At stage 27, handβ expression was detected in branchial areas and in the heart **(B10–B12)**. At stage 30, handβ expression was retained in branchial areas and the heart, and was also detected in the ventral region of the caudal fin **(B13–B16)**.

At stage 25, *handα* was expressed in the ventral branchial mesenchyme surrounding the endostyle (Figure 2A8), the medial stripe of the lower-lip mesenchyme and cardiac region (Figure 2A9), and the caudal region (Figure 2A10). At stage 27, expression was detected in branchial areas and in the heart (Figures 2A11–A13). By the latest stage examined (stage 30), *handα* expression was retained only in the cardiac region (Figure 2A14).

*Handβ* expression was first detected during head-process formation (stages 22–23) in the ventral mesenchyme of the head process (Figures 2B1–B6). At stage 25, *handβ* was expressed in the ventral branchial mesenchyme surrounding the endostyle (Figure 2B7) and in the cardiac region (Figure 2B8). A weaker signal was also observed in the medial stripe of the lower-lip mesenchyme. Unlike *handα*, *handβ* was not detected in the caudal region at this stage (Figure 2B9).

At stage 27, *handβ* expression intensified in the branchial areas and heart (Figures 2B10–B12) and persisted in both regions through stage 30 (Figures 2B13, B15, B16). At stage 30, an additional *handβ* domain appeared in the ventral caudal fin (Figures 2B13, B14).

No specific handγ signal was detected by whole-mount ISH under the conditions and developmental stages examined. Sequence analysis nevertheless confirmed that the probe corresponds to a distinct handγ genomic locus.

### Expression of *hand1* and *hand2* during shark *C. griseum* embryogenesis

At the earliest stages examined (stages 24–25; Ballard et al., 1993) of shark *C. griseum* embryogenesis, *hand1* was expressed along the entire dorsoventral extent of all branchial arches, although the signal was relatively weak and diffuse and accompanied by background staining (Figure 3A). Additional expression was detected in the otic vesicle, in sensory ganglia, and in the cloacal region.

**FIGURE 3:**
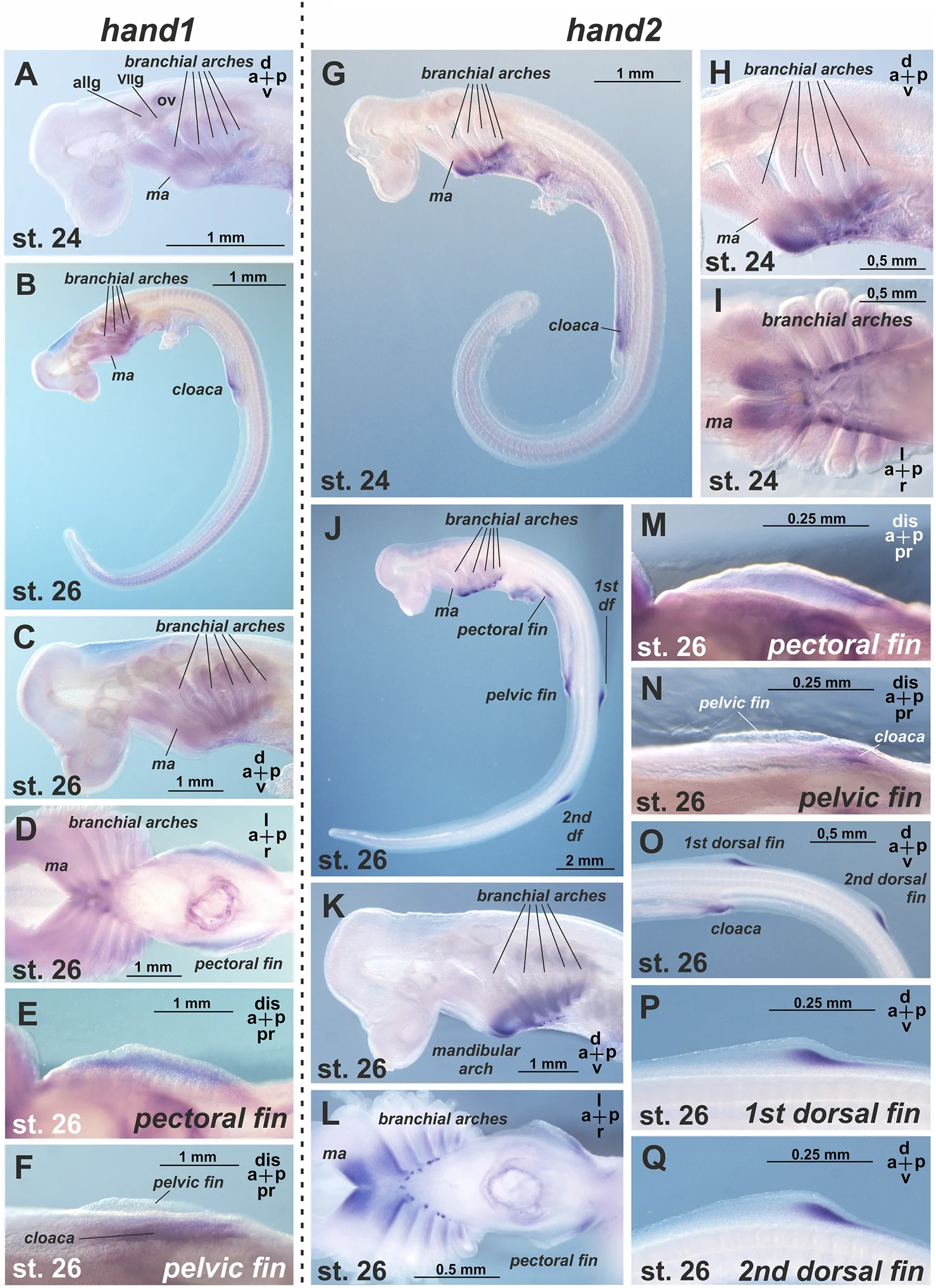
Expression of *hand1* and *hand2* in *C*. *griseum* embryos at stages 24–26 (staged according to Ballard et al., 1993). Whole-mount ISH patterns are shown for the branchial region and developing fins. For *hand1*, staining at the earliest stages was comparatively weak and accompanied by diffuse background; statements concerning undetected domains therefore refer specifically to the whole-mount ISH conditions used. *Hand2* produced a stronger and more spatially restricted signal, including posterior domains of paired and dorsal fins. Scale bars and panel-specific anatomical labels are shown in the figure. At stage 24, *hand1* is expressed along the full dorsoventral extent of all branchial arches, with a weak, diffuse signal accompanied by background staining **(A)**. At stage 26, expression appears in the pectoral fins and occupies most of the fin mesenchyme, but no specific *hand1* signal is detected in the dorsal fins under the whole-mount ISH conditions used **(C–E)**. Expression in the branchial arches and cloacal region remains diffuse **(C,D,F)**. *Hand2* is first detected at stage 24 and produces a strong, well-defined signal with little detectable background. Expression is confined to the ventral regions of all branchial arches, with the strongest signal in the mandibular arch and bright punctate domains at the ventral bases of the arches. Expression is also detected in the cloacal region **(G–I)**. At stage 26, *hand2* appears in the posterior mesenchyme of the first and second dorsal fins and in the posterior pectoral fin mesenchyme **(J–Q)**. allg–anterior lateral line ganglion, ma–mandibular arch, ov–otic vesicle, VIIg–VII nerve ganglion.

At stage 26, *hand1* expression appeared in the pectoral fins and encompassed much of the fin mesenchyme (Figures 3B–F). No specific *hand1* signal was detected in the dorsal fins under the whole-mount ISH conditions used, whereas staining in the branchial arches and cloacal region remained comparatively weak and diffuse. By stage 27, *hand1* expression was also detected in the pelvic fins and became more distally restricted in paired fins (Supplementary Figure S3).

*Hand2* expression was also first detected at stages 24–25, but its distribution differed markedly from that of *hand1* (Figure 3). *Hand2* produced a strong, well-defined signal with little detectable background. In the branchial arches, expression was confined to ventral regions and was strongest in the mandibular arch. Bright punctate domains were present at the ventral base of each arch. Like *hand1*, *hand2* was also expressed in the cloacal region.

At stage 26, *hand2* expression appeared in the posterior mesenchyme of the first and second dorsal fins and in the posterior mesenchyme of pectoral fin (Figure 3). By stage 26.5, expression had intensified in all branchial arches and in the dorsal and pectoral fins. In the pectoral fin, the domain encompassed nearly all of the mesenchyme except its anterior-most region, while a weak signal appeared in the posterior pelvic fin. At stage 27, *hand2* was strongly expressed in the posterior mesenchyme of the dorsal, pectoral, and pelvic fins (Supplementary Figure S3). At stages 28–29, expression persisted in the branchial arches and all fins, while the punctate domains at the ventral bases of the arches gradually weakened (Figure 4; Supplementary Figure S4). By stages 29–30, the fin domains had contracted to posterodistal regions distal to the radials, and the ventral punctate domains in the arches had almost disappeared (Supplementary Figure S4).

**FIGURE 4:**
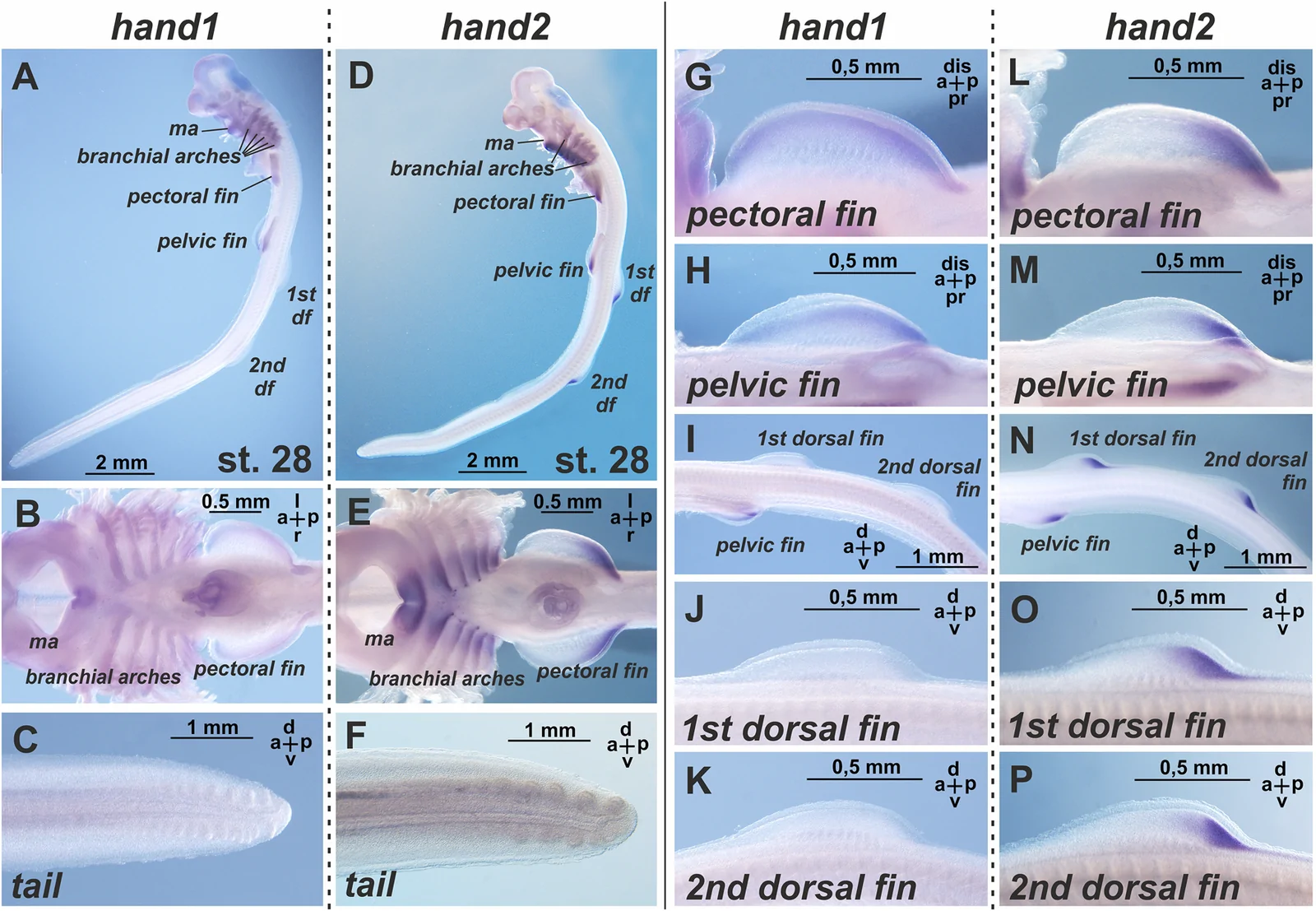
Expression of *hand1* and *hand2* in *C*. *griseum* embryos at stage 28 (staged according to Ballard et al., 1993). *hand1* and *hand2* show distinct spatial distributions in paired fins and the branchial region, whereas *hand2* additionally shows a posterior domain in dorsal fins. Panel-specific views and anatomical labels are indicated in the figure. At stage 28, *hand1* expression in the pectoral and pelvic fins is confined to the distal mesenchyme and extends along the anteroposterior extent of both paired fins. Expression is also detected in the mandibular symphysis and branchial arches **(A–C)**, **(G–K)**. No expression was detected in the dorsal fins under the conditions examined **(J–K)**. *Hand2* is expressed in the branchial arches and in the dorsal, pectoral, and pelvic fins. In the fins, the signal is concentrated in the posterior mesenchyme. The punctate domains at the ventral bases of the branchial arches are weaker than at earlier stages **(D–F)**, **(L–P)**. df – dorsal fin, ma – mandibular arch.

Together, these observations reveal marked differences in the spatiotemporal expression of the two *hand* paralogs in shark. *Hand1* was expressed along the full dorsoventral extent of the branchial arches and in the distal mesenchyme across the anteroposterior extent of the paired fins, but no specific *hand1* domain was detected in the dorsal fins by whole-mount ISH; by stage 30, specific fin-associated *hand1* signal was detected only in the posterior pelvic fin mesenchyme under the whole-mount ISH conditions used. *Hand1* was also transiently expressed in the otic vesicle, in sensory ganglia, and in the mandibular symphysis. By contrast, *hand2* expression was first detected in the dorsal and pectoral fins at stage 26 and subsequently in the pelvic fins, with expression concentrated in the posterior and, at later stages, posterodistal mesenchyme. Because the *hand1* staining was weaker, more diffuse, and accompanied by background staining at stages 24–29, fine-scale comparisons of signal boundaries should be interpreted cautiously.

To complement the whole-mount ISH observations, we analyzed *hand1* and *hand2* transcript levels in dissected *C. griseum* fins by qRT-PCR (Supplementary Figure S5; Supplementary Tables S5–S6). In the pooled samples analyzed, *hand1* transcript abundance in the dorsal fin was approximately 22-fold lower than in the pectoral fin and 24-fold lower than in the pelvic fin, whereas *hand2* transcript abundance in the dorsal fin was approximately 1.6-fold lower than in the pectoral fin and was similar in the pectoral and pelvic fins. Low levels of *hand1* transcripts were thus detectable in the dorsal fin by qRT-PCR despite the absence of a specific whole-mount ISH signal.

## Discussion

### Evolution of the *hand* gene family in vertebrates: evidence from phylogenetics and genomic synteny

The phylogenetic analysis supports the identification of the experimentally characterized lamprey and shark Hand sequences while emphasizing the limited resolution of deeper paralog relationships. *Lampetra fluviatilis* Handα, Handβ and Handγ consistently group with their respective *P. marinus* counterparts, whereas alternative assignments of cyclostome Handα and Handβ to gnathostome Hand1 or Hand2 cannot be statistically distinguished by AU topology tests. Accordingly, the phylogeny supports stable within-cyclostome α/β/γ correspondences but does not by itself establish one-to-one orthology between cyclostome and gnathostome *Hand* paralogs.

The local genomic synteny analysis provides complementary, but more limited, information. Within gnathostomes, *hand1* and *hand2* are associated with distinct and partly conserved local contexts, including the paralogous *sap30l-galnt10* and *sap30-galnt7* combinations. Corresponding *galnt-sap-hand* regions were previously analysed in human, mouse and chicken (Viiri et al., 2009). In cyclostomes, however, the neighborhoods of *handα*, *handβ* and *handγ* show only partial correspondence to gnathostome loci. The current data are therefore compatible with an ancient relationship among duplicated *hand*-containing genomic regions but do not independently establish one-to-one orthology across the cyclostome-gnathostome split.

The presence of multiple *Hand* copies in both cyclostomes and gnathostomes is consistent with the complex history of vertebrate genome duplication, including lineage-specific polyploidization and subsequent rediploidization (Marlétaz et al., 2024; Yu et al., 2024). However, the present study does not attempt chromosome-scale reconstruction of ancestral linkage groups, and the specific duplication history of individual Hand copies cannot be inferred from local synteny alone. The phylogeny, topology tests, and local genomic context are therefore interpreted together, with the deeper orthology of cyclostome and gnathostome paralogs remaining unresolved.

### Divergence of *hand* paralog expression in cyclostomes and gnathostomes

Our data show that at least two hand paralogs show detectable expression during lamprey embryogenesis. Earlier studies treated lamprey *Hand* expression as if it represented a single gene (Cerny et al., 2010; Häming et al., 2011; Kuraku et al., 2010). Comparison with the revised sequence dataset indicates that the gene examined by Kuraku et al. (2010) corresponds to the α lineage, whereas that examined by Cerny et al. (2010) corresponds to the β lineage. Consequently, the composite expression pattern previously attributed to lamprey Hand encompasses contributions from distinct paralogs with partially overlapping but different spatiotemporal domains.

Both lamprey paralogs are expressed in the heart and ventral branchial region, recapitulating *Hand* domains described previously in lampreys. However, *handα* is activated substantially earlier and has a broader early domain, whereas *handβ* persists at later stages and acquires an additional domain in the ventral caudal fin. This partitioning is compatible with regulatory partitioning after duplication, but expression data alone do not demonstrate functional subfunctionalization. The *handβ* caudal-fin domain is particularly notable because it extends *hand* deployment to a median fin in a jawless vertebrate. No specific *handγ* signal was detected under the conditions examined. The sequence-verified probe maps to a distinct *handγ* genomic locus and contains an uninterrupted *hand*-like coding frame in the region analysed; therefore the negative ISH result does not establish that *handγ* is a pseudogene. It could reflect low or spatially restricted expression, expression outside the examined stages or tissues, transcriptional silencing, or pseudogenization not demonstrable from the present data.

The two shark paralogs also show different expression patterns. *Hand2* is detected in the ventral part of the branchial arches and posterior mesenchyme of paired (pectoral and pelvic) and median (dorsal) fins. *Hand1* gives a broader but weaker branchial signal and is detected in paired fins, whereas no specific *hand1* signal was detected in dorsal fins under the whole-mount ISH conditions used. These observations demonstrate paralog-specific expression differences but, in the absence of functional perturbation, are not interpreted as evidence of functional divergence.

The shark fin-bud patterns partly resemble those reported in other gnathostomes, but direct species comparisons require caution. In the pectoral fin of *S. canicula*, Onimaru et al. (2015) reported a broad *hand1* expression domain extending through approximately the anterior two-thirds of the fin bud, whereas *hand2* was restricted more posteriorly. This spatial separation was interpreted in the context of anterior–posterior patterning of the chondrichthyan fin, in which several gene-expression boundaries are shifted relative to the tetrapod limb. Our observations in *C. griseum* are consistent with a spatial distinction between *hand1* and *hand2* expression domains, but reveal a somewhat different distribution of *hand1*, which becomes associated with the distal mesenchyme of the paired fins. Thus, the precise spatial deployment of *hand1* does not appear to be identical between the two shark species. This difference should be interpreted cautiously, since the studies differ in developmental sampling: *S. canicula hand1* expression was characterized primarily at stage 30, whereas our analysis follows *C. griseum* expression across a broader developmental series. Moreover, Onimaru et al. focused on anterior–posterior patterning of the pectoral fin and did not provide a comparable analysis of *Hand* paralogs in dorsal fins. Together, these observations suggest that spatial differentiation of *hand1* and *hand2* expression is shared between the two chondrichthyans, whereas the precise deployment of these paralogs within the fin mesenchyme may be more variable. In *C. griseum*, the absence of a detectable specific *hand1* domain in the dorsal fin by whole-mount ISH, together with the low transcript level detected by qRT-PCR, further indicates that this difference concerns the spatial distribution and relative abundance of expression rather than an absolute presence or absence of *hand1* transcription.

### Evolutionary implications for branchial and fin development

Comparison of lamprey and shark expression confirms and extends previous evidence that Hand deployment in ventral and branchial regions predates the cyclostome–gnathostome split (Cerny et al., 2010; Kuraku et al., 2010; Onimaru et al., 2011). However, whole-mount expression does not establish the germ-layer or cell-lineage identity of the *Hand*-positive branchial mesenchyme, so direct homology of specific branchial cell populations between lamprey and shark is not inferred here.

In sharks and other gnathostomes, both *hand* paralogs are expressed in the branchial apparatus and paired fins. This shared deployment is compatible with proposed developmental links between branchial arches and paired appendages, including modern versions of Gegenbaur’s hypothesis (Gillis et al., 2013; Gillis and Hall, 2016; Sleight and Gillis, 2020; Wen et al., 2026). However, overlapping expression of developmental regulators is consistent with the involvement of shared regulatory components but does not, by itself, demonstrate shared developmental competence or serial homology.

The expression of *hand2* in both paired and median fins is also consistent with recruitment of components of an evolutionarily older median-fin program into paired appendages (Freitas et al., 2006; 2014). Shared deployment of the Shh regulatory module in paired and median fins provides stronger evidence for such regulatory repurposing (Letelier et al., 2018; Hawkins et al., 2021; Zdral et al., 2026). Conversely, the preferential detection of spatially distinct *hand1* expression domains in paired rather than dorsal fins suggests differential deployment of this paralog among fin types, rather than strict paired-fin specificity. A comparable partitioning of expression domains has been described for *shox* and *shox2* (Ermakova et al., 2025).

Taken together, the observed *Hand* expression patterns are compatible with the idea that paired-appendage development incorporated regulatory components also used in other mesodermal structures, but they do not discriminate among competing homology models on their own.

The most direct contribution of the present study is the resolution of distinct cyclostome *Hand* paralogs, the identification of a lamprey *handβ* caudal-fin domain, and the comparative description of paralog-specific *Hand* expression across paired and median fins in a cartilaginous fish.

## Materials and methods

### Animals and sample preparation

All animal procedures were approved by the Institutional Animal Care and Use Committee of the Shemyakin–Ovchinnikov Institute of Bioorganic Chemistry (Moscow, Russia; protocol IACUC 229, 1 February 2018) and were conducted in accordance with applicable institutional requirements and local legislation.

Sexually mature European river lampreys (*Lampetra fluviatilis*) were collected in the Leningrad Region, Russia. Embryos were obtained by artificial fertilization in the laboratory and staged according to Tahara (1988).

*Chiloscyllium griseum* eggs and embryos were obtained in collaboration with the scientific department of the Moskvarium Center for Oceanography and Marine Biology (Moscow, Russia) and staged according to Ballard et al. (1993).

*Lampetra fluviatilis* and *C. griseum* were selected as representatives of Cyclostomata and Chondrichthyes, respectively, because sufficient numbers of embryos were available for experimental analysis.

For ISH, embryos were fixed in MEMFA (3.7% formaldehyde, 100 mM MOPS, 2 mM EGTA, and 1 mM MgSO4), dehydrated in methanol, and stored at −20 °C.

Total RNA was purified from pooled *C. griseum* embryos (three embryos per preparation) using the innuPREP RNA Mini Kit 2.0 (Analytik Jena, Berlin, Germany).

### Phylogenetic and local genomic synteny analyses

Hand homologs were identified using the NCBI BLAST web server (Sayers et al., 2025) and the BLAST + suite (Altschul et al., 1990; Camacho et al., 2009).

Translated nucleotide searches were performed with TBLASTN (Gertz et al., 2006) through the NCBI BLAST web server.

Searches included the nucleotide collection (nr/nt) and whole-genome shotgun contigs (WGS) databases.

Protein sequences were aligned with MUSCLE. The phylogenetic workflow was implemented in IQ-TREE (Minh et al., 2020), with model selection using ModelFinder (Kalyaanamoorthy et al., 2017). The initial alignment comprised 708 positions; poorly represented columns were removed using a reproducible occupancy criterion retaining sites with≤50% gaps, yielding 204 positions in the trimmed alignment.

Maximum-likelihood phylogenetic analyses were performed in IQ-TREE 3.1.4. ModelFinder was used to evaluate amino-acid substitution models, and Q. INSECT + F + I + G4 was selected by the Bayesian information criterion for the trimmed dataset.

Branch support was evaluated using 1,000 SH-like approximate likelihood-ratio test (SH-aLRT) replicates and 1,000 ultrafast bootstrap (UFBoot) replicates. To assess robustness to model heterogeneity, an additional analysis was performed using the site-heterogeneous LG + C60 + F + G4 model with posterior mean site frequencies (PMSF).

The principal Hand-family tree was treated as unrooted. Rooting sensitivity was evaluated by repeating the C60/PMSF analysis with a divergent *B. floridae* Twist-like bHLH sequence. Alternative one-to-one orthology scenarios were evaluated using constrained maximum-likelihood trees under Q. INSECT + F + I + G4 and the approximately unbiased (AU) test implemented in IQ-TREE. The unconstrained topology and four constrained hypotheses placing cyclostome Handα or Handβ with gnathostome Hand1 or Hand2 were compared using 10,000 RELL bootstrap replicates. Sequence datasets, alignments, tree files and topology-test output are provided as Supplementary Data.

Local genomic synteny analysis was used as a complementary approach to protein phylogeny. Hand loci were identified in available genome assemblies using annotated gene records and BLAST searches, and their immediate genomic neighborhoods were inspected manually using NCBI genomic records. The analysis was intended to compare conservation and divergence of local genomic context rather than to reconstruct chromosome-scale paralogons or ancestral linkage groups. Because genome assembly and annotation quality varies among taxa, absence of a neighboring gene from the local maps was not by itself interpreted as evidence of gene loss. The recent chromosome-level *L. fluviatilis* assembly kcLamFluv1.1 (GCF_964198595.1) was additionally searched for the three experimentally characterized *hand* loci; genomic positions and source records are summarized in Supplementary Table S2. A chromosome-level genome assembly was not available for *C. griseum*; therefore, genomic-neighborhood comparisons for chondrichthyans relied on available annotated assemblies of related cartilaginous fishes.

For *P*. *marinus*, local genomic-neighborhood analysis was performed using the chromosome-level germline reference assembly UKy_Petmar_22M1. pri1.0 (RefSeq accession GCF_048934315.1).

### Cloning and sequence verification of *hand* fragments and whole-mount *in situ* hybridization

Fragments corresponding to *L. fluviatilis handα*, *handβ*, and *handγ* were amplified by PCR from cDNA preparations synthesized from total RNA with the following gene-specific primers:

LF_Handα_Frw1; ATG​AGC​GTC​GCT​GGC​GGC​TT;

LF_Handα_Frw2; CCG​CTC​GCC​TCG​CTC​TGC​AG;

LF_Handα_Rev1; TCG​AGT​GCC​CAC​ACG​TGC​TG;

LF_Handα_Rev2; CCT​TTG​CTC​TTC​CTC​TCG​GC;

LF_Handβ_Frw1; ATG​AGT​CTG​GTG​GGT​GGC​TA;

LF_Handβ_Frw2; CTC​ACC​ACC​ACC​CGG​TCA​TG;

LF_Handβ_Rev1; CGT​TTC​AAC​TCC​TGA​GCC​CA;

LF_Handβ_Rev2; CCA​GTC​CGT​CCC​CGC​TTC​TT;

LF_Handγ_Frw1; ATG​TTC​CTA​CAG​CAG​GGC​CA;

LF_Handγ_Frw2; CCT​GTC​TCA​CCG​CCC​ATT​CC;

LF_Handγ_Rev1; ACA​TTG​GCA​CTG​AAC​CCT​GT;

LF_Handγ_Rev2; CCT​CAG​CTC​CGA​GAA​GGC​GA.

*C. griseum hand1* and *hand2* cDNAs were amplified by PCR using the following primers:

CG_Hand1_full_Frw1; GAA​GTG​AAT​CTA​GTT​GAG​GA;

CG_Hand1_Frw2; ATT​ATG​AGC​CTA​GTG​GGG​AGT​TT;

CG_Hand1_Rev1; AAC​TGT​GCA​AAT​TTG​CAA​GT;

CG_Hand1_Rev2; AAT​TCA​CTG​GTT​CAG​CTC​GAG​AG;

CG_Hand2_Frw1; AAG​TCG​CCT​TAC​ACG​CGG​AA;

CG_Hand2_Rev1; ATC​TTC​CAA​AGT​TCA​CGT​TC;

CG_Hand2_Frw2; ATT​ATG​AGT​TTA​GTT​GGA​GGG​TT;

CG_Hand2_Rev2; AAT​CTC​ACT​GTT​TGA​GCT​CCA​GAG.

Nested PCR was performed with the Encyclo PCR kit (Evrogen, Moscow, Russia), using the Frw1/Rev1 primer pair in the first round and Frw2/Rev2 in the second.

Amplified fragments were cloned into the pAL2-T vector (Evrogen), and sequence identity was verified by Sanger sequencing of independent clones. The experimentally characterized *L. fluviatilis handα* and *handβ* and *C. griseum hand1* and *hand2* transcript-derived sequences have been deposited in GenBank under accession numbers QB134029 (*L. fluviatilis* handα), QB134030 (*L. fluviatilis* handβ), QB134031 (C. griseum hand1), and QB134032 (C. griseum hand2); the sequence-verified *L. fluviatilis* handγ genomic fragment was deposited under accession number QB134033.

Whole-mount ISH was performed as described by Ermakova et al. (2024b).

Probes were used at 2 μg/mL. Before hybridization, shark embryos were incubated for 30 min at room temperature in 0.4% hydrogen peroxide, 0.5× SSC, and 5% formamide under fluorescent illumination with continuous agitation.

Probe identities and boundaries were established from the Sanger-sequenced cloned inserts (Supplementary Table S4). Pairwise comparisons showed global nucleotide identities of 54.8% for *L. fluviatilis handα/handβ*, 49.9% for *handα/handγ*, 51.4% for *handβ/handγ*, and 65.7% for the target-derived portions of *C. griseum hand1*/*hand2* probes. The longest exact target-derived matches were 26, 10, 12, and 16 nt, respectively. Common engineered cloning/primer tails were excluded from the shark paralog-specificity comparison.

Prehybridization and hybridization were performed at 57 °C for 3 and 16 h, respectively.

Post-hybridization washes were performed at 57 °C in hybridization buffer (two 30-min washes), followed by a 1:1 mixture of hybridization buffer and 2× SSC containing 0.1% Tween-20 (two 30-min washes). Embryos were then washed at the hybridization temperature in 2× SSC containing 0.1% Tween-20 (five 10-min washes), followed by 0.2× SSC containing 0.1% Tween-20 (two 20-min washes).

Composition of the hybridization buffers:

- Lamprey hybridization buffer: 50% formamide, 4× SSC, 200 μg/mL Torula RNA, 0.2% Tween-20, 0.5% CHAPS, 5 mM EDTA, 100 μg/mL heparin sulfate; pH 7.4.
- Shark hybridization buffer: 50% formamide, 4× SSC, 1 mg/mL Torula RNA, 1× Denhardt’s solution, 0.2% Tween-20, 0.1% CHAPS, 5 mM EDTA; pH 7.4.

For each species, developmental stage, and probe, *hand* expression was examined in at least five embryos. A pattern was considered reproducible when observed in at least 80% of the embryos analyzed.

After staining, lamprey embryos were cleared through a glycerol:PBS series to 70% glycerol and photographed.

Images were acquired with a Leica M205 stereomicroscope.

Positive and negative technical controls were included in the whole-mount ISH experiments. Previously characterized *foxg1β* (Ermakova et al., 2024a) and *chordin-like1* (Ermakova et al., 2025) probes were used as positive controls for *L. fluviatilis* and *C. griseum*, respectively. Additional control probes included *nogginD* in lamprey (Ermakova et al., 2020), which produces weak diffuse staining, and *CYSTLR2-like* (NCBI Gene ID: 122554946) in shark, which did not produce a specific spatial expression domain under the conditions used. Representative control experiments are shown in Supplementary Figure S6.

### Quantitative RT-PCR (qRT-PCR)

Total RNA was isolated from pectoral, pelvic and dorsal fins and control body-wall tissue of *C. griseum* using the innuPREP RNA Mini Kit 2.0 (Analytik Jena, Jena, Germany). Fin tissues were dissected from *C. griseum* embryos at stages 29–30. For each tissue type, the available RNA represented a single pooled biological sample comprising the corresponding tissues collected from two embryos. RNA concentration was measured using a Qubit® fluorometer (Invitrogen), and RNA integrity was assessed by agarose gel electrophoresis. First-strand cDNA was synthesized using the MMLV RT kit (SK021; Evrogen, Russia). Gene-specific primers were synthesized by Evrogen (Moscow, Russia). qPCR was performed using 5× qPCRmix-HS SYBR (PK147; Evrogen, Russia) on a DTprime real-time PCR instrument (DNA-Technology, Russia), with three technical replicates for each reaction. EF1α and ODC1 were used as reference genes. Relative expression levels were calculated using the ΔΔCq method, with the mean Cq of the two reference genes used for normalization and pectoral-fin expression used as the calibrator. Primer sequences are provided in Supplementary Table S5.

## Data Availability

The original contributions presented in the study are included in the article/Supplementary Material, further inquiries can be directed to the corresponding authors.
