# Supplementary material for: The hand of evolution: conservation and divergence of hand paralogs in cyclostomes and gnathostomes
Source: Front Cell Dev Biol. 2026 Oct 29;14:1944613. doi: 10.3389/fcell.2026.1944613 (PMC13756586; doi:10.3389/fcell.2026.1944613)
Supplement: Supplementary file 2 [file Supplementaryfile1.docx]

**Supplementary Information**

**The hand of evolution: conservation and divergence of *hand* paralogs in cyclostomes and gnathostomes**

Galina V. Ermakova, Irina V. Meyntser, Alexandr V. Kucheryavyy, Andrey G. Zaraisky, Andrey V. Bayramov

**Supplementary Data**

**Data S1a**: exact protein FASTA dataset used for the principal 76-taxon Hand phylogeny.

**Data S1b**: *B. floridae* Twist-like sequence used for outgroup sensitivity.

**Data S2a**: complete MUSCLE alignment (77 taxa, 708 positions, including Twist sensitivity taxon).

**Data S2b**: principal trimmed alignment (76 taxa, 204 positions).

**Data S3a**: principal C60/PMSF unrooted tree file.

**Data S3b**: Twist-containing sensitivity tree file.

**Data S3c**: IQ-TREE topology-test output.

**Supplementary Table S1. AU topology tests of alternative cyclostome-gnathostome Hand orthology hypotheses**

| **Topology** | **AU P value** | **Interpretation** |
| --- | --- | --- |
| Unconstrained | 0.554 | not rejected |
| Handα -> Hand1 | 0.432 | not rejected |
| Handα -> Hand2 | 0.458 | not rejected |
| Handβ -> Hand1 | 0.629 | not rejected |
| Handβ -> Hand2 | 0.121 | not rejected |

**Supplementary Table S2. Genome assemblies and experimentally relevant lamprey *Hand* loci**

| **Species / assembly** | **Locus** | **Genomic placement** | **Identification** | **Notes** |
| --- | --- | --- | --- | --- |
| *Lampetra fluviatilis* / kcLamFluv1.1 (GCF_964198595.1) | *handα* | chr15, OZ243577.1 | sequence-based | immediate neighborhood incompletely annotated |
| *Lampetra fluviatilis* / kcLamFluv1.1 (GCF_964198595.1) | *handβ* | LOC144937656; XM_078865043.1 | annotated locus | most directly annotated Lampetra Hand locus |
| *Lampetra fluviatilis* / kcLamFluv1.1 (GCF_964198595.1) | *handγ* | chr5, OZ243567.1 | exact match to 622-bp probe | immediate neighborhood incompletely annotated |
| *Petromyzon marinus* | *handα*/β/γ | UKy_Petmar_22M1.pri1.0 (GCF_048934315.1) | annotation + sequence search | chromosome-level germline reference assembly |

**Supplementary Table S3. Sequence inventory for Hand phylogenetic analyses**

| **Tree label** | **Species code** | **Hand designation** | **Sequence accession** | **Length (aa)** | **Status** |
| --- | --- | --- | --- | --- | --- |
| NeVe Hand2 | NeVe | Hand2 | XP_001632876.2 | 145 | Database protein sequence |
| NeVe Hand1 | NeVe | Hand1 | XP_032237799.1 | 184 | Database protein sequence |
| AsRu Hand2-like | AsRu | Hand2-like | XP_033632101.1 | 239 | Database protein sequence |
| AcPl Hand2-like | AcPl | Hand2-like | XP_022098957.1 | 248 | Database protein sequence |
| SaKo Hand2-like | SaKo | Hand2-like | XP_006814979.1 | 207 | Database protein sequence |
| BrFl Hand2-like | BrFl | Hand2-like | XP_035682283.1 | 235 | Database protein sequence |
| CiIn TF protein | CiIn | TF protein | NP_001071729.1 | 378 | Database protein sequence |
| PeMa Handα | PeMa | Handα | XP_032811338.2 | 312 | Database protein sequence |
| PeMa Handγ | PeMa | Handγ | XP_075930595.1 | 322 | Database protein sequence |
| PeMa Handβ | PeMa | Handβ | XP_032812432.2 | 294 | Database protein sequence |
| MyGl Hand2-like a | MyGl | Hand2-like a | XP_067970451.1 | 199 | Database protein sequence |
| MyGl Hand2-like b | MyGl | Hand2-like b | XP_067988228.1 | 231 | Database protein sequence |
| CaMi Hand2a | CaMi | Hand2a | XP_007894139.1 | 212 | Database protein sequence |
| CaMi Hand2b | CaMi | Hand2b | XP_007906292.1 | 196 | Database protein sequence |
| ChPl Hand2 | ChPl | Hand2 | XP_043560436.1 | 212 | Database protein sequence |
| ChPl Hand2-like | ChPl | Hand2-like | XP_043560083.1 | 197 | Database protein sequence |
| HeOc Hand2 | HeOc | Hand2 | XP_060698927.1 | 212 | Database protein sequence |
| HeOc Hand1-like | HeOc | Hand1-like | XP_060692902.1 | 197 | Database protein sequence |
| ScCa Hand2 | ScCa | Hand2 | XP_038661713.1 | 213 | Database protein sequence |
| ScCa Hand1-like | ScCa | Hand1-like | XP_038649412.1 | 198 | Database protein sequence |
| RhTy Hand2 | RhTy | Hand2 | XP_020388998.1 | 212 | Database protein sequence |
| RhTy Hand1-like | RhTy | Hand1-like | XP_048459759.1 | 197 | Database protein sequence |
| StTi Hand2 | StTi | Hand2 | XP_048382488.2 | 211 | Database protein sequence |
| StTi Hand2-like | StTi | Hand2-like | XP_048399645.1 | 197 | Database protein sequence |
| CaCa Hand2 | CaCa | Hand2 | XP_041042150.1 | 212 | Database protein sequence |
| CaCa Hand1-like | CaCa | Hand1-like | XP_041048766.1 | 196 | Database protein sequence |
| AmRa Hand2 | AmRa | Hand2 | XP_032874210.1 | 214 | Database protein sequence |
| AmRa Hand1-like | AmRa | Hand1-like | XP_032885915.1 | 194 | Database protein sequence |
| LeEr Hand2 | LeEr | Hand2 | XP_055488288.1 | 214 | Database protein sequence |
| LeEr Hand1-like | LeEr | Hand1-like | XP_055498522.1 | 195 | Database protein sequence |
| PrPe Hand2 | PrPe | Hand2 | XP_051876099.1 | 211 | Database protein sequence |
| PrPe Hand1-like | PrPe | Hand1-like | XP_051870638.1 | 194 | Database protein sequence |
| AcRu Hand1-like a | AcRu | Hand1-like a | XP_034782480.2 | 192 | Database protein sequence |
| AcRu Hand1-like b | AcRu | Hand1-like b | XP_033877359.1 | 192 | Database protein sequence |
| AcRu Hand2 a | AcRu | Hand2 a | XP_033871718.1 | 208 | Database protein sequence |
| AcRu Hand2 b | AcRu | Hand2 b | XP_033891859.1 | 208 | Database protein sequence |
| PoSp Hand2 | PoSp | Hand2 | XP_041115350.1 | 208 | Database protein sequence |
| PoSp Hand2-like | PoSp | Hand2-like | XP_041079472.1 | 208 | Database protein sequence |
| PoSp Hand1-like a | PoSp | Hand1-like a | XP_041122759.1 | 192 | Database protein sequence |
| PoSp Hand1-like b | PoSp | Hand1-like b | XP_041078488.1 | 192 | Database protein sequence |
| PoSe Hand2 | PoSe | Hand2 | XP_039606015.1 | 209 | Database protein sequence |
| PoSe Hand1-like | PoSe | Hand1-like | XP_039631370.1 | 191 | Database protein sequence |
| ErCa Hand2 | ErCa | Hand2 | XP_028646997.1 | 209 | Database protein sequence |
| ErCa Hand1-like | ErCa | Hand1-like | XP_028668777.1 | 191 | Database protein sequence |
| LeOc Hand2 | LeOc | Hand2 | XP_006630021.1 | 208 | Database protein sequence |
| LeOc Hand1 | LeOc | Hand1 | XP_006632012.1 | 190 | Database protein sequence |
| MeCy Hand2 | MeCy | Hand2 | XP_036372678.1 | 204 | Database protein sequence |
| MeCy Hand2-like | MeCy | Hand2-like | XP_036406946.1 | 203 | Database protein sequence |
| MeCy Hand1-like | MeCy | Hand1-like | XP_036378851.1 | 189 | Database protein sequence |
| ScFo Hand2 | ScFo | Hand2 | XP_018591281.1 | 204 | Database protein sequence |
| ScFo Hand2-like | ScFo | Hand2-like | XP_018593936.2 | 190 | Database protein sequence |
| ScFo Hand1 | ScFo | Hand1 | XP_018584020.1 | 196 | Database protein sequence |
| DaRe Hand2 | DaRe | Hand2 | NP_571701.3 | 205 | Database protein sequence |
| LaCh Hand2 | LaCh | Hand2 | XP_005993037.1 | 210 | Database protein sequence |
| LaCh Hand1 | LaCh | Hand1 | XP_005988374.1 | 196 | Database protein sequence |
| PrAn Hand2 | PrAn | Hand2 | XP_043917896.1 | 206 | Database protein sequence |
| PrAn Hand1 | PrAn | Hand1 | XP_043925637.1 | 201 | Database protein sequence |
| XeTr Hand2 | XeTr | Hand2 | NP_001093695.1 | 210 | Database protein sequence |
| XeTr Hand1 | XeTr | Hand1 | NP_001016743.1 | 194 | Database protein sequence |
| AnCa Hand2 | AnCa | Hand2 | XP_062837444.1 | 247 | Database protein sequence |
| AnCa Hand1 | AnCa | Hand1 | XP_008112985.2 | 229 | Database protein sequence |
| PyBi Hand2 | PyBi | Hand2 | XP_015743874.1 | 164 | Database protein sequence |
| PyBi Hand1 | PyBi | Hand1 | XP_015747100.2 | 150 | Database protein sequence |
| CarCar Hand2 | CarCar | Hand2 | XP_048702952.2 | 215 | Database protein sequence |
| CarCar Hand1 | CarCar | Hand1 | XP_048718349.1 | 197 | Database protein sequence |
| GaGa Hand2 | GaGa | Hand2 | NP_990297.2 | 216 | Database protein sequence |
| GaGa Hand1 | GaGa | Hand1 | XP_046756248.1 | 201 | Database protein sequence |
| MuMu Hand2 | MuMu | Hand2 | NP_034532.3 | 217 | Database protein sequence |
| MuMu Hand1 | MuMu | Hand1 | NP_032239.1 | 216 | Database protein sequence |
| HoSa Hand2 | HoSa | Hand2 | NP_068808.1 | 217 | Database protein sequence |
| HoSa Hand1 | HoSa | Hand1 | NP_004812.1 | 215 | Database protein sequence |
| LaFl Handα | LaFl | Handα | QB134029 | 310 | Experimental sequence added in this revision |
| LaFl Handβ | LaFl | Handβ | QB134030 | 302 | Experimental sequence added in this revision |
| LaFl Handγ | LaFl | Handγ | QB134033 | 322 | Experimental genomic sequence added in this revision; protein reconstruction provisional. |
| ChGr Hand1 | ChGr | Hand1 | QB134031 | 197 | Experimental sequence added in this revision |
| ChGr Hand2 | ChGr | Hand2 | QB134032 | 212 | Experimental sequence added in this revision |

Note. GenBank accession numbers for the newly characterized experimental sequences are QB134029 (*L. fluviatilis handα*), QB134030 (*L. fluviatilis handβ*), QB134031 (*C. griseum hand1*), QB134032 (*C. griseum hand2*), and QB134033 (*L. fluviatilis handγ* genomic fragment).

**Supplementary Table S4. Sequence-level specificity of the ISH probes**

| **Comparison** | **Global identity** | **Ungapped-overlap identity** | **Longest perfect match** | **Exact tract** |
| --- | --- | --- | --- | --- |
| *handα* vs *handβ* | 54.8% | 72.0% | 26 nt | GACACGAAGCTGTCCAAGATCAAGAC |
| *handα* vs *handγ* | 49.9% | 68.1% | 10 nt | GAGCATCAAC |
| *handβ* vs *handγ* | 51.4% | 63.5% | 12 nt | CAGAGCATCAAC |
| *C. griseum* *hand1* vs *hand2* | 65.7% | 72.8% | 16 nt | AGAGCATCAACAGCGC |

For the shark comparison, common engineered primer/cloning sequence was excluded. Probe coordinates, primer combinations and complete sequence records are provided with the sequence data package.

**Supplementary Figure S1. Outgroup-sensitivity phylogeny of Hand/Hand-like proteins.**

Maximum-likelihood analysis including a divergent *Branchiostoma floridae* Twist-like bHLH sequence, performed using the site-heterogeneous LG+C60+F+G4/PMSF model in IQ-TREE. The tree is graphically rooted on the *B. floridae* Twist-like sequence to illustrate the effect of outgroup rooting. Inclusion of this divergent sequence does not alter the well-supported clustering of the experimentally characterized *L. fluviatilis* Handα, Handβ and Handγ sequences with the corresponding *P. marinus* sequences, or the placement of the *C. griseum* Hand1 and Hand2 sequences within the respective gnathostome groups. Selected ultrafast bootstrap (UFBoot) support values based on 1,000 replicates are shown at nodes relevant to the placement of the experimentally characterized sequences and representative deeper relationships. Background shading indicates the taxonomic affiliation of the sequences and is used for visualization only; it does not denote phylogenetically supported clades. The long branch separating the Twist-like sequence from the Hand/Hand-like proteins precludes confident inference of the biological root of the Hand family; the principal Hand-family phylogeny is therefore presented as unrooted in Figure 1. Branch lengths represent substitutions per site.

**Supplementary Figure S2. Local genomic synteny of *Hand* genes in representative chordates.**

Local genomic neighborhoods surrounding *hand1* and *hand2* loci are shown for representative gnathostomes, together with the genomic neighborhoods of cyclostome *handα*, *handβ*, and *handγ* genes and the single *Hand*-like locus of lancelet *B. floridae*. Genes are represented by arrows indicating their relative orientation; chromosome or linkage-group assignments are indicated where available. The *hand1* and *hand2* loci occur in distinct but partially conserved genomic contexts across gnathostomes. In particular, the *hand1–sap30L–galnt10* and *hand2–sap30–galnt7* combinations correspond to paralogous local genomic regions previously described in vertebrates (Viiri et al., 2009). Selected similarities between cyclostome and gnathostome *Hand*-associated neighborhoods are highlighted by yellow boxes. Cyclostome Hand loci retain subsets of genes present in gnathostome *Hand*-associated neighborhoods, including *cep44*, *hmgb2*, and *galnt7* near *handα*, but these local similarities do not by themselves establish one-to-one orthology between cyclostome *handα/β/γ* and gnathostome *hand1/hand2*. Gene symbols follow the annotations of the respective genome assemblies and are presented in lowercase for graphical consistency; lineage-specific paralog designations, such as *hmgb2a*, are retained. The figure represents local genomic synteny comparisons and is not intended as a reconstruction of chromosome-scale paralogons or ancestral linkage groups.

**Abbreviations:** *cnot8*, CCR4-NOT transcription complex subunit 8; *faxdc2*, fatty acid hydroxylase domain containing 2; *larp1*, La ribonucleoprotein 1; *hpgd*, 15-hydroxyprostaglandin dehydrogenase; *sap30L*, SAP30-like; *sap30*, Sin3A-associated protein 30; *galnt10*, polypeptide N-acetylgalactosaminyltransferase 10; *galnt7*, polypeptide N-acetylgalactosaminyltransferase 7; *mfap3*, microfibril-associated protein 3; *glra3*, glycine receptor alpha 3; *cep44*, centrosomal protein 44; *fbxo8*, F-box protein 8; *scrg1*, scrapie responsive gene 1; *hmgb2/hmgb2a*, high mobility group box 2/2a; *galntl6*, polypeptide N-acetylgalactosaminyltransferase-like 6; *gria1*, glutamate ionotropic receptor AMPA type subunit 1; *prmt9*, protein arginine methyltransferase 9; *tmem184*, transmembrane protein 184; *usp1*, ubiquitin-specific peptidase 1; *trappc2-like*, trafficking protein particle complex 2-like.

**Supplementary Figure S3.** **Expression of *hand1* and *hand2* in *C. griseum* embryos at stage 27** (staged according to Ballard et al., 1993).

The figure documents the distribution of both paralogs in branchial and fin regions at stage 27.

At stage 27, *hand1* is expressed in the distal mesenchyme along the entire anteroposterior extent of the pectoral and pelvic fins, while no specific *hand1* signal is detected in the dorsal fins under the whole-mount ISH conditions used. Diffuse expression is also observed in the branchial arches and the cloacal region (A, B, E–I).

*hand2* is expressed in the posterior mesenchyme of the pectoral, pelvic, and dorsal fins. Expression is maintained predominantly in the ventral branchial arches (C, D, J–N).

**Supplementary Figure S4.** **Expression of *hand1* and *hand2* in *C. griseum* fins at stages 29-30** (staged according to Ballard et al., 1993).

Whole-mount ISH is shown for paired and dorsal fins. The lack of visible *hand1* staining in a fin domain is interpreted as no detectable specific signal under the conditions used rather than as proof of complete transcript absence.

At stages 29–30, *hand1* expression in the paired fins is weaker than at earlier stages (A–F, L–P). By stage 30, no specific *hand1* signal is detected in the pectoral fins under the whole-mount ISH conditions used, whereas a signal is retained in the posterior pelvic fin (L).

*hand2* expression is maintained in paired and dorsal fins, but its domains contract to posterodistal regions distal to the radials (G–K, Q–U).

**Targeted qRT-PCR validation of fin expression**

Targeted qRT-PCR was used as an orthogonal validation of the fin expression patterns observed by whole-mount ISH. Total RNA was isolated from pectoral, pelvic and dorsal fins and control body-wall tissue from *C. griseum* embryos at stages 29–30 (after Ballard et al., 1993) using the innuPREP RNA Mini Kit 2.0 (Analytik Jena, Jena, Germany). For each tissue type, the available RNA represented a single pooled biological sample comprising the corresponding tissues collected from two embryos. RNA concentration was measured using a Qubit® fluorometer (Invitrogen), and RNA integrity was assessed by agarose gel electrophoresis. First-strand cDNA was synthesized using the MMLV RT kit (SK021; Evrogen, Russia). Gene-specific primers were synthesized by Evrogen (Moscow, Russia). qPCR was performed using 5× qPCRmix-HS SYBR (PK147; Evrogen, Russia) on a DTprime real-time PCR instrument (DNA-Technology, Russia), with three technical replicates for each reaction. *EF1α* and *ODC1* were used as reference genes. Relative expression levels were calculated using the ΔΔCq method, with the mean Cq of the two reference genes used for normalization and pectoral-fin expression used as the calibrator. Primer sequences are provided in Supplementary Table S5.

**Supplementary Table S5. Primer pairs used for targeted qRT-PCR validation.**

| **Gene** | **Primer** | **Sequence (5′→3′)** | **Amplicon** |
| --- | --- | --- | --- |
| *hand1* | H1_RT_F1 | ACACCTTGTCCAAGGACAGT | 127 bp |
| *hand1* | H1_RT_R1 | CTTCTGGTCGAGCCGTTTAC | 127 bp |
| *hand2* | H2_RT_F1 | TGGAGGGTTCCCTCATCATC | 105 bp |
| *hand2* | H2_RT_R1 | TGGAAGTACGGGCTCTCTTC | 105 bp |
| *EF1α* | EF1a_RT_F1 | TGGAAGATCAACCGTAAGGA | 121 bp |
| *EF1α* | EF1a_RT_R1 | AGACATCTTGCAGAGGAAGA | 121 bp |
| *ODC1* | ODC1_RT_F1 | AGGCTCAGCAATGACTTGAA | 117 bp |
| *ODC1* | ODC1_RT_R1 | GGCTTTCCTGGTTCAGAAGA | 117 bp |

**Supplementary Table S6. qRT-PCR Cq measurements and descriptive normalized expression.**

| **Tissue** | **EF1α Cq** | **ODC1 Cq** | ***hand1* Cq** | ***hand2* Cq** | ***hand1* relative** | ***hand2* relative** |
| --- | --- | --- | --- | --- | --- | --- |
| Control | 14.3; 15.4; 15.2 | 18.2; 19.1; 18.8 | 31.8; 32.5; 32.6 | 31.4; 31.8; 31.8 | 0.0058 | 0.0044 |
| Pectoral fins | 15.2; 16.1; 15.8 | 18.5; 18.9; 19.1 | 24.6; 25.8; 25.5 | 23.9; 24.7; 24.2 | 1.0000 | 1.0000 |
| Pelvic fins | 17.4; 17.5; 18.3 | 20.6; 21.2; 21.1 | 26.8; 27.6; 27.3 | 26.1; 26.7; 26.4 | 1.1100 | 0.9660 |
| Dorsal fins | 16.1; 16.6; 16.7 | 19.1; 19.6; 19.8 | 29.8; 30.8; 30.8 | 25.2; 26.0; 25.8 | 0.0458 | 0.6230 |

Values in the four Cq columns are the three technical qPCR replicates from the same pooled biological sample. Relative expression was calculated from mean technical-replicate Cq values after normalization to the mean Cq of *EF1α* and *ODC1*; pectoral fin was set to 1. These technical replicates were not treated as independent biological replicates, and no inferential statistics were performed.

**Supplementary Figure S5. Targeted qRT-PCR validation of *hand1* and *hand2* expression in *C. griseum* fins.**

Relative transcript levels were measured in control body-wall tissue and pectoral, pelvic and dorsal fins. For each tissue, RNA was available from one pooled biological sample comprising corresponding tissue from two embryos, and qPCR measurements were performed in three technical replicates. Expression was normalized to *EF1α* and *ODC1*, with pectoral-fin expression set to 1. *hand1* transcript abundance was markedly lower in dorsal fin than in either paired fin, whereas *hand2* remained readily detectable in dorsal fin and differed much less between paired and dorsal fins. Low *hand1* transcript was detectable in dorsal fin by qRT-PCR despite the lack of a specific whole-mount ISH signal. Because only one pooled biological sample per tissue was available, the data are presented as targeted orthogonal validation of the ISH observations and were not subjected to inferential statistical analysis.

**Supplementary Figure S6. Representative positive and negative control probes used in whole-mount ISH experiments.**

In L. fluviatilis, foxg1β was used as a positive control and shows a characteristic spatially restricted expression pattern (Ermakova et al., 2024), whereas nogginD produces weak diffuse staining without a comparable discrete expression domain (Ermakova et al., 2020). In C. griseum, chordin-like1 was used as a positive control and shows strong spatially restricted expression, including the branchial arches (Ermakova et al., 2025), whereas a probe corresponding to the *C. griseum* ortholog of the *C. plagiosum* *cysteinyl leukotriene receptor 2-like* gene (CYSTLR2-like; NCBI Gene ID: 122554946) does not produce a specific spatial expression domain under the same whole-mount ISH conditions.
